# Expert opinion on the management of pain in hospitalised older patients with cognitive impairment: a mixed methods analysis of a national survey

**DOI:** 10.1186/s12877-015-0056-6

**Published:** 2015-04-29

**Authors:** Kirsty TM Rodger, Corinne Greasley-Adams, Zoe Hodge, Emma Reynish

**Affiliations:** Department of Geriatric Medicine, NHS Fife, Victoria Hospital, Hayfield Road, Kirkcaldy, KY2 5AH UK; School of Applied Social Science, University of Stirling, Colin Bell Building, Stirling, FK9 4LA UK; Acute Pain Service, NHS Fife, Victoria Hospital, Hayfield Road, Kirkcaldy, KY2 5AH UK

**Keywords:** Pain, Dementia, Cognitive impairment, Analgesia, Safety

## Abstract

**Background:**

Hospitalised older patients are complex. Comorbidity and polypharmacy complicate frailty. Significant numbers have dementia and/or cognitive impairment. Pain is highly prevalent. The evidence base for pain management in cognitively impaired individuals is sparse due to methodological issues. A wealth of expert opinion is recognised potentially providing a useful evidence base for guiding clinical practice. The study aimed to gather expert opinion on pain management in cognitively impaired hospitalised older people.

**Methods:**

Consultant Geriatricians listed as dementia leads in the National Dementia Audit were contacted electronically and invited to respond. The questionnaire sought information on their role, confidence and approach to pain management in cognitively impaired hospitalised patients. Responses were analysed using a mixed methods approach.

**Results:**

Respondents considered themselves very confident in the clinical field. Awareness of potential to do harm was highly evident. Unequivocally responses suggested paracetamol is safe and should be first choice analgesic, newer opiates should be used preferentially in renal impairment and nefopam is unsafe. A grading of the safety profile of specific medications became apparent, prompting requirement for further evaluation and holistic assessment.

**Conclusion:**

The lack of consensus reached highlights the complexity of this clinical field. The use of paracetamol first line, newer opiates in renal impairment and avoidance of nefopam are immediately transferrable to clinical practice. Further review, evaluation and comparison of the risks associated with other specific analgesics are necessary before a comprehensive clinical guideline can be produced.

**Electronic supplementary material:**

The online version of this article (doi:10.1186/s12877-015-0056-6) contains supplementary material, which is available to authorized users.

## Background

Older patients in hospital are complex with co-morbidity, polypharmacy and frailty being the norm. Pain is highly prevalent. Although a subjective and personal experience, pain is unanimously unpleasant with sensory and emotional factors impacting on functional and mental capacity, social interaction and quality of life [1]. Multiple physiological and cognitive factors such as damaged nociceptive pathways, lower pain thresholds and mood contribute to the prevalence of pain in the elderly population. With more disease (especially osteoarthritis), immobility, muscle weakness and frequent falls persistent pain is common, particularly chronic musculoskeletal pain. When pain is coupled with cognitive impairment the impact and disability is far greater [2].

Pain is no less frequent or intense in people with dementia, with around 50% of these individuals experiencing pain regularly [3]. However studies have shown that pain is often under recognised and under treated in the cognitively impaired [2,4]. People with dementia are prescribed and given less analgesia than other older people [5]. When analgesia is prescribed to someone with dementia 83% do not receive their medication [6] and 76% of people with dementia did not receive regular analgesia post operatively for hip repair, despite 42% expected to be in severe pain [7].

People with dementia may have difficulty in communicating their pain and often care staff do not recognise the non-verbal signs they may exhibit when in pain, hence missing diagnostic clues [8]. Furthermore, reduction of semantic memory for pain seen in people with dementia and reduced ability to identify pain is associated with a decline in pain reporting [9].

Pain may manifest itself differently in the cognitively impaired and associated changes in behaviour may be attributed to something other than pain leading to late or misdiagnosis of pain [10,11]. The behavioural and psychiatric symptoms of dementia are typically treated with antipsychotics or sedatives which may be unnecessary and harmful if the underlying stimulus such as pain is recognised and treated accordingly. Ultimately untreated pain is devastating in these individuals unable to explain their pain [12]. Using a standardised protocol to treat pain in nursing home residents with moderate to severe dementia has been demonstrated to significantly improve pain, agitation and aggression and could help reduce the unnecessary use of antipsychotics in people with dementia [13].

Management of pain in older people and particularly the cognitively impaired is challenging. Adverse drug reactions are two to three times more common in older people than younger individuals [14]. The risk of delirium is higher and further augmented in those with existing cognitive impairment whether due to vascular or degenerative brain disease. Additionally, lack of recognition of side effects in individuals with dementia may occur [15].

Despite recent publications regarding pain management in older people such as that in Age and Ageing [16], there is little specifically targeted at those with cognitive impairment. It is accepted that clinical guidance should be based on the best available evidence and that from randomised controlled trials is gold standard. Due to the complexity and heterogeneity of this elderly population with cognitive impairment randomised controlled trials are methodologically difficult, hence the evidence base in this group is lacking. However we recognise the wealth of expert opinion in this field. The aim of this paper is to collate this opinion in order for it to be useful in clinical practice.

## Methods

We obtained a list of consultant Geriatricians/Dementia Leads identified in the National Dementia Audit from the British Geriatrics Society. This amounted to one hundred contacts. We contacted them electronically with an invitation to respond to a questionnaire relating to their role, experience, confidence and opinions on pain management in elderly patients with cognitive impairment. A second and third electronic message was sent after two weeks and one month respectively as a reminder. Their responses were anonymised. Ethical approval was sought but the Local Research Ethics Committee advised review was not necessary.

Those completing the questionnaire were presented with three case scenarios (Additional file 1). They were asked to provide an account of how they would manage the case and what factors needed to be taken into consideration in the management of such cases.

Analysis was undertaken in two parts. Responses to closed questions (individuals’ confidence with the management of each case scenario and their use of specific analgesic medications) were analysed quantitatively. Thematic analysis was undertaken of text responses. Comments were grouped under common themes and reported findings based on the common responses provided. The coding framework used was established from the responses and was not pre-determined.

This work was supported by NHS Fife Endowments-Geriatric Medicine Training Fund. This specifically funded analysis and interpretation of data.

## Results

### Respondents

From the 88 individuals successfully contacted, 42 accessed and completed at least some part of the survey (47.7% response rate). 5 were omitted from analysis due to failure to complete the survey. Thus 37 responses were included in the analysis. 73% of the respondents worked at consultant level, 78% considered themselves to be specialists in the management of delirium and 27% were involved in a memory or specialist dementia clinic.

In all cases over 85% of respondents rated themselves at 7 or above on a scale from 1 (not confident) to 10 (very confident) when asked to self-rate their confidence in managing each case (Additional file 2).

### Responses

There was no definite consensus of opinion on the management of each case or use of individual medication. Respondents were aware of the potential to do harm when prescribing medication and management suggestions were cautious and exhaustive. Respondents’ replies showed a number of key themes. These key themes will now be discussed individually.

### Use of analgesic medication

#### Response to case studies

In the free text replies regarding management of the three case scenarios, there was overwhelming agreement that paracetamol (orally/intravenously) should be the first step in pain management.

All respondents reported that the principles of the WHO pain ladder should be followed for subsequent prescribing. The existence of further differentiation in steps of the pain ladder (i.e. in the strength of analgesia to be prescribed next) was not recognised with suggestions including oxycodone, oramorph, codeine, buprenorphine, tramadol, NSAIDS and fentanyl. With each suggestion respondents highlighted the need to consider side effects and “balancing the need for analgesia with the risk of opiate induced delirium”. In one case scenario treatment with oxycodone was consensual due to comorbid chronic kidney disease. Specific comments pertained to codeine and its unpredictable metabolism. Using codeine alongside paracetamol (in preference to co-codamol) was considered preferable, allowing more control over medication if side effects occurred. In some cases despite canvasing opinion from specialists who were highly confident in managing each scenario, respondents’ opinions were completely in opposition to each other e.g. whether to consider NSAIDS or not.

In addition to noting the potential for serious side effects respondents were also aware of the importance of differing routes of administration, noting topical analgesia is well tolerated. Also regular rather than PRN prescribing of analgesia was deemed beneficial due to poor self-reporting of pain.

#### Response to use of specific medication

**Figure 1:**
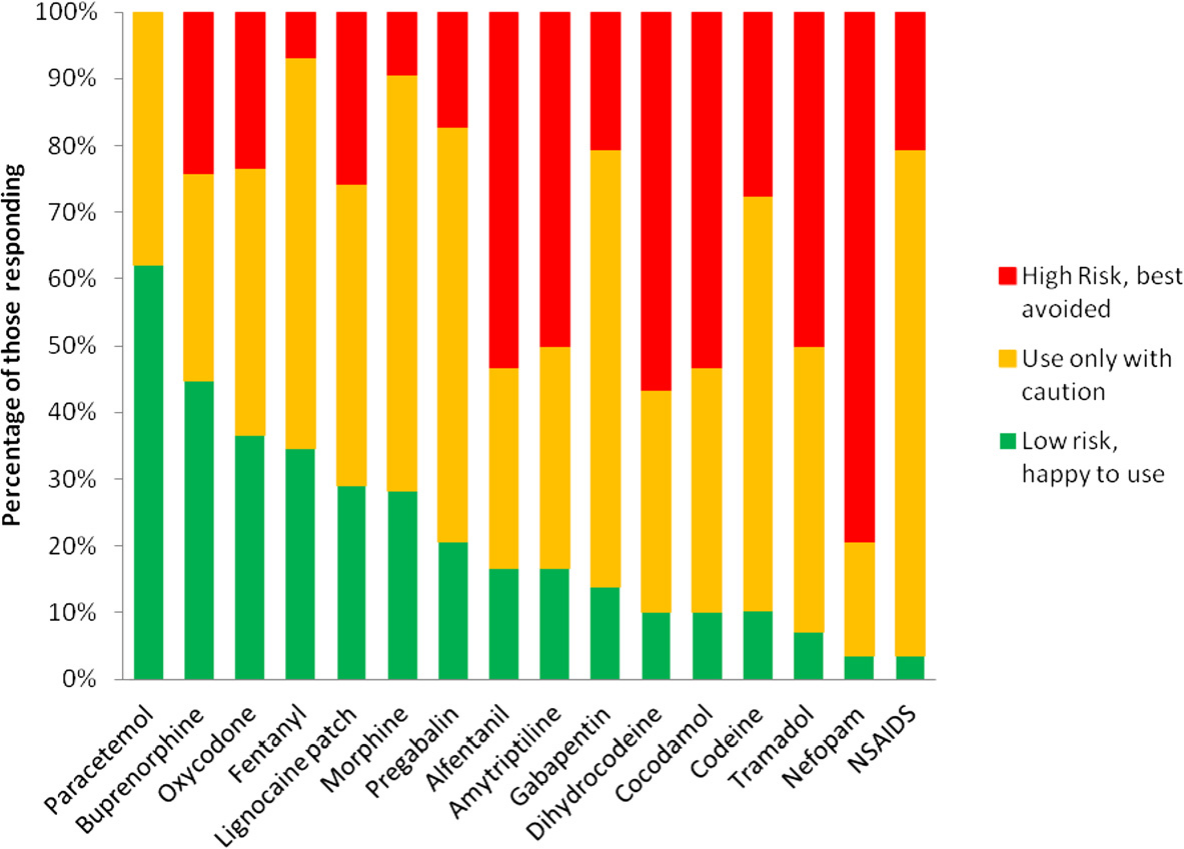
Medication use by respondents in older people with cognitive impairment.

When responding to the section of questionnaire examining use of specific medications the caution exhibited in prescribing at the top end of the ladder was also in evidence but a grading of the safety profile of individual medications became apparent (Figure 1).

Specific points raised by respondents included overwhelming agreement that paracetamol should be the first step in pain management since its efficacy is well evidenced. It’s paucity of side effects was noted, in particular it was stated that it has no impact on cognition and is well tolerated.

The weak opiates; co-codamol (avoided completely by 53%, used only with caution by 37%), codeine (avoided completely by 28%, used only with caution in 64%) and Dihydrocodeine (avoided by 57%, used only with caution by 33%) were generally unpopular. Respondents recognised that they often elicited unwanted side effects with effects on cognitive and bowel function of particular concern. If used, close monitoring and low dose titration was recommended.

50% of respondents stated they would avoid using the opiate Tramadol completely and 43% would only use with extreme caution, recognising its potential to precipitate delirium.

The majority (91%) did suggest using morphine sulphate. However they noted caution in prescribing due to renal excretion and suggested checking renal function prior to initiation and dose titrated to effect.

Treatment with oxycodone was considered to be a viable option by the majority (77%). In their opinion it had a favourable side effect profile compared to morphine sulphate. Respondents particularly noted fewer problems with accumulation of metabolites in patients with renal impairment and it was felt to be less likely to precipitate delirium.

Buprenorphine patches were considered a safe alternative to oral opioids and regarded as “renally friendly” but slow onset of action hampered use in the acute setting.

Use of NSAIDs was only advocated with extreme caution (76%) or avoided completely (21%). Under certain conditions they should only be prescribed for a short period and with PPI cover. Topical use was also considered.

Since amitriptyline is “not well tolerated even at low doses” it was avoided by the majority (50%) and used only with caution by 33%. It was noted to be particularly useful for neuropathic pain but if used, only with caution due to its high risk of causing delirium.

Of the gabapentinoids Pregablin was favoured over Gabapentin. Low slow gradual dose titration was recommended but again it was recognised that the class may precipitate sedation and confusion.

Lidocaine plasters would be considered by the majority (74%) for localised pain. There was conflicting opinion of evidence base for non post herpetic neuralgia and it was noted prescription restrictions apply in many areas of the UK.

Fentanyl patches were also considered in patients who demonstrated tolerance to equi-analgesic doses of other opioids. Doses otherwise were felt to be too high for most patients in this frail population. Toxicity was felt to be a significant risk.

There was overwhelming agreement (80%) that nefopam should not be used as an analgesic in this patient population. Reasons for this included its anticholinergic properties making it “notoriously deliriogenic”.

### Need for further exploration

Over 70% of respondents in all cases highlighted the need for further assessment and investigation. Suggested requests included: more background and medical history for the patient, identification of the cause of pain, identification of delirium and treatment of its underlying cause, multi-disciplinary assessment, early involvement of family/caregivers, discussion of ongoing/anticipatory care, consultation with specialist teams e.g. pain or palliative care and the need to monitor pain and re-assess regularly.

### Avoidance of harm

Over half of respondents provided comments coded under this category. There was clear awareness of potential side effects of pain medications. Suggestions pointed the need to review and monitor patients for these especially by screening and observing for delirium and opiate toxicity but additionally by considering the impact of prescriptions on renal and bowel function. The need to review existing medications was highlighted with recommendations to prevent accumulation and toxicity e.g. digoxin, or of stopping some medication during the intercurrent illness causing the pain e.g. withholding anti-hypertensives peri-operatively to prevent acute kidney injury. There were also suggestions of medications to be co-prescribed to prevent side effects being experienced e.g. gastric protection in the use of NSAIDs. Maintenance of hydration was also highlighted as essential to protect renal and cardiac function when treating pain.

### Need for monitoring and review

34 respondents (91.8%) commented that assessment and re-assessment of pain was crucial. Approximately one third of respondents (11) used clinical assessment alone but others used established pain assessment tools e.g. ABBEY (11), Doloplus (2), PAINAID (1), local (1).

The final themes highlighted the importance of the environment and maintenance of overall health using the principles of comprehensive geriatric assessment. This included consideration of underlying cause of falls, screening for other causes of delirium and treating accordingly, maintaining homeostasis of existing conditions, taking opportunity to rationalise existing medications and reduce burden of polypharmacy.

## Discussion

The management of pain is a fundamental part of the systematic and effective delivery of compassionate and dignified care to older people. The experience of pain can be disabling both physically and psychologically. This is heightened in those with cognitive impairment and/or dementia, yet high quality evidence to guide management in this area is lacking. The synthesis of expert opinion in this study highlights the multi-faceted complexity of pain management in this population and significant potential for harm as a result of the analgesic smorgasbord currently available to the clinician. The preferences of respondents in this study do reflect the principles outlined in the BGS standard [16] although it only considered the management of pain in older people in general not specifically those with cognitive impairment. It also recognised the lack of well controlled studies in this population from which to base the evidence.

This study has many strengths; opinion was gathered from experts across the UK. There was no pressure or incentive for them to respond other than assisting the common goal of improving patient care. The response rate to the questionnaire was 48% and the majority of respondents were very confident in the management of these scenarios when asked to self-rate their confidence. A mixed methods analysis was employed to capture the nuances of individual clinicians approach to pain management. Limitations of the study however do include the fact that results are based on expert opinion i.e. the lowest grade of evidence, but as stated previously there is an evidence gap in the management of pain in this population. Despite the good response rate the total number of respondents (37) is small.

## Conclusions

The lack of consensus that has resulted from this study highlights the complexity of the clinical field when managing pain in this population. The need for evaluation of the whole individual (a Comprehensive Geriatric Assessment approach), including their family and caregivers and the environment are promoted rather than an approach that would just simply treat the pain. The opinion that the potential to do significant harm has come out loud and clear in all responses: some medications are more likely to cause harm than others and therefore evident that some are a more favourable option than others, so despite no definite consensus the appearance of higher risk and lower risk medications has become apparent. Steps 2 and 3 on the WHO analgesic ladder are blurred in this high risk population. The principles guiding the management of pain set out in the WHO pain ladder are applicable but as severity of pain increases, the choice of analgesic becomes more difficult because of the facility to do harm. A number of results are however unequivocal; paracetamol is safe and should be the first choice analgesic in this situation. Newer opiates should be used in preference in renal impairment. There appeared to be caution noted with prescription of codeine and tramadol. Nefopam appears unsafe for use in this population. These points are immediately transferrable to clinical practice but further unpicking and comparison of the risks associated with other specific analgesics in this population is necessary before a comprehensive clinical guideline can be produced. The principles guiding pain management in this population however are that individuals should be comprehensively assessed and prescription of analgesia should be done with a view to minimising resulting harm; using the principle of “start low and go slow”.

## Additional files

Additional file 1:
**Case scenarios included in questionnaire.**
Additional file 2:
**Case scenario 1, 2 and 3 confidence levels.**
