# Supplementary material for: Expert opinion on the management of pain in hospitalised older patients with cognitive impairment: a mixed methods analysis of a national survey
Source: BMC Geriatr. 2015 Apr 29;15:56. doi: 10.1186/s12877-015-0056-6 (PMC4419491; doi:10.1186/s12877-015-0056-6)
Supplement: Additional file 1: — Case scenarios included in questionnaire. [file 12877_2015_56_MOESM1_ESM.docx]

**Additional file 1: Case Scenarios included in questionnaire**

**Case 1**: 85 year old man with mild Alzheimer’s disease. Admitted with fall and resulting fractured neck of femur. Experiences mild to moderate pain

**Case 2**: 87 year old woman with infected chronic leg ulcers being conservatively managed by vascular surgeons. Incident delirium, moderate to severe pain, multiple comorbidities including Chronic Kidney Disease stage 4 & polypharmacy

**Case 3**: 93 year old with known dementia. Admitted following a fall. Fractured humerus, delirious, unable to take normal diet or medication.
