# Supplementary figures and images for: Expert opinion on the management of pain in hospitalised older patients with cognitive impairment: a mixed methods analysis of a national survey
Source: BMC Geriatr. 2015 Apr 29;15:56. doi: 10.1186/s12877-015-0056-6 (PMC4419491; doi:10.1186/s12877-015-0056-6)

**Additional file 2: Case Scenario 1, 2 and 3 Confidence Levels**

**
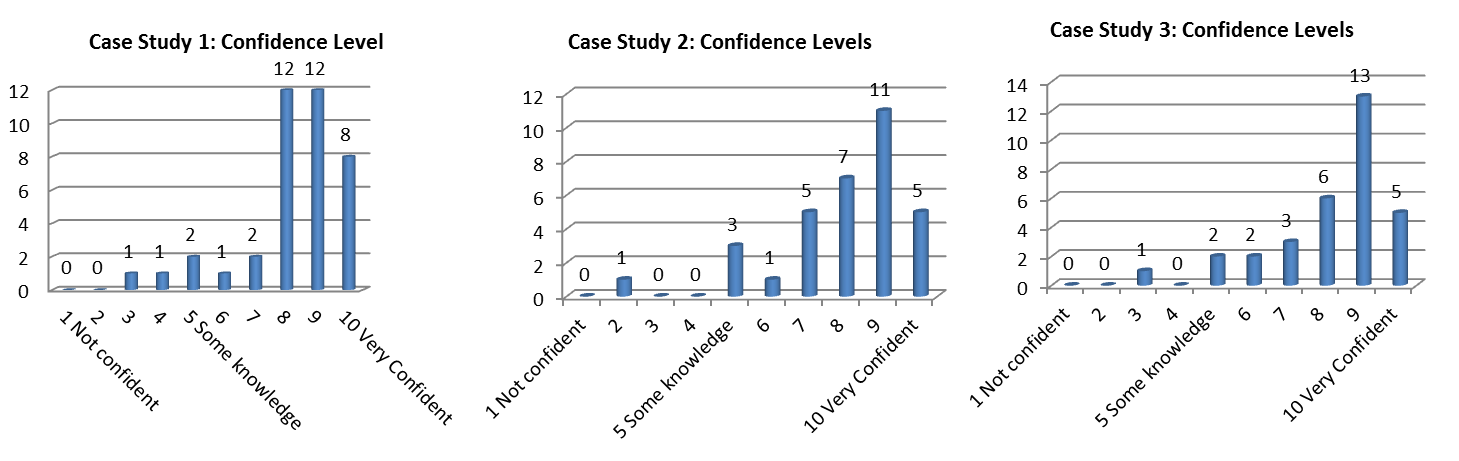
**

Supplement: Additional file 2: — Case scenario 1, 2 and 3 confidence levels. [file 12877_2015_56_MOESM2_ESM.docx]
